# Adverse outcomes associated with recorded victimization in mental health electronic records during the first UK COVID-19 lockdown

**DOI:** 10.1007/s00127-022-02393-w

**Published:** 2022-11-24

**Authors:** Giouliana Kadra-Scalzo, Daisy Kornblum, Robert Stewart, Louise M. Howard

**Affiliations:** 1 Institute of Psychiatry, Psychology and Neuroscience, King’s College London, BRC Nucleus, IOPPN, Mapother House, De Crespigny Park, London, UK; 2 South London and Maudsley NHS Foundation Trust, London, UK

**Keywords:** COVID-19, Mental health, Adverse outcomes, Victimisation, Domestic violence

## Abstract

**Purpose:**

The impact of COVID-19 pandemic policies on vulnerable groups such as people with mental health problems who experience violence remains unknown. This study aimed to investigate the prevalence of victimization recorded in mental healthcare records during the first UK lockdown, and associations with subsequent adverse outcomes.

**Methods:**

Using a large mental healthcare database, we identified all adult patients receiving services between 16.12.2019 and 15.06.2020 and extracted records of victimisation between 16.03.2020 and 15.06.2020 (first UK COVID-19 lockdown). We investigated adverse outcomes including acute care, emergency department referrals and all-cause mortality in the year following the lockdown (16.06.2020- 01.11.2021). Multivariable Cox regressions models were constructed, adjusting for socio-demographic, socioeconomic, clinical, and service use factors.

**Results:**

Of 21,037 adults receiving mental healthcare over the observation period, 3,610 (17.2%) had victimisation mentioned between 16.03.2020 and 15.06.2020 (first UK COVID-19 lockdown). Service users with mentions of victimisation in their records had an elevated risk for all outcomes: acute care (adjusted HR: 2.1; 95%CI 1.9–2.3, *p* < 0.001), emergency department referrals (aHR: 2.0; 95%CI 1.8–2.2; *p* < 0.001), and all-cause mortality (aHR: 1.5; 95%CI 1.1–1.9; *p* = 0.003), when compared to service users with no recorded victimisation. We did not observe a statistically significant interaction with gender; however, after adjusting for possible confounders, men had slightly higher hazard ratios for all-cause mortality and emergency department referrals than women.

**Conclusion:**

Patients with documented victimisation during the first UK lockdown were at increased risk for acute care, emergency department referrals and all-cause mortality. Further research is needed into mediating mechanisms.

## Background

Although the impact of the COVID-19 outbreak on individual, societal and global level is still emerging, what we do know is that it has had an unprecedented impact on mental health services [1]. In the period following the first lockdown in the UK, these included early discharges from inpatient units [2], reduced outpatient appointments [3], moving of therapies to online delivery [4], and cancellations of appointments [3]. A UK survey of mental health care staff found that early in the COVID-19 pandemic, mental health professionals were specifically concerned about people experiencing domestic abuse and family conflict [5]. Subsequently, clinical communities have called for urgent action to better understand the impact of COVID-19 restrictions on vulnerable groups such as people with existing mental health problems and those who have experienced violence such as domestic abuse [6]. Evidence from prior research examining patterns from pandemics such as Ebola and Zika virus has reported specific concerns about the impact of restrictive measures on domestic violence [7]. In April 2020, the World Health Organization Europe reported there was a 60% increase in emergency calls from women reporting intimate partner violence as compared to the same period last year [8]. A similar pattern has been observed globally, in countries such as Australia [9], USA [10], China [11], and Norway [12].

There is already a substantial body of evidence indicating that men and women with pre-existing mental illnesses are at a significantly increased risk of being victims of all forms of violence as compared to the general population [13, 14], with 15–45% of patients reporting experiences of victimization in the past year, and 40–90% reporting lifetime victimization [13]. Domestic violence (DV) has been reported to be especially prevalent amongst mental health service users, with 27% of women and 13% of men with serious mental illnesses (SMI) reporting experiencing DV in the past year, compared to 9 and 5%, respectively, in general population samples [15]. Victimization and DV are associated with substantial healthcare and personal cost. Direct medical and mental healthcare costs approximate £1,730 million per annum in the UK [16]. On an individual level, amongst mental health service users the experience of victimization has been associated with physical health problems, poorer functioning and quality of life, drug and/or alcohol use and perpetration of crime [13, 17, 18]. Clinically, victimization has been associated with greater symptom severity, depression and anxiety, and increased hospitalization [19]. Furthermore, compared to those victims without SMI, female victims with SMI are significantly more likely to report adverse psychological effects (92 vs. 64%) and attempted suicide (53 vs. 3%) [15]. Finally, approximately 68% of mental health service users have reported such experiences to a mental health professional [14], which suggests that mental health services can be a critical point of contact for many experiencing victimization and/or domestic violence. To the best of our knowledge, this is one of the first studies which has aimed to [1] investigate the prevalence of victimization mentions extracted from Electronic Health Records (EHRs) in mental health services during the first UK COVID-19 lockdown and [2] establish the associations between such mentions and the adverse outcomes of (a) acute care, (b) emergency department referrals and (c) all-cause mortality in the year following the lockdown. Furthermore, in keeping with previous recommendation [20] for best practice in this area of research, we stratified our findings by gender.

## Methods

### Settings

We assembled a retrospective cohort study using secondary data from the mental health EHRs of the South London and Maudsley NHS Foundation Trust (SLAM). SLAM is one of the largest providers of secondary healthcare in Europe, serving four London boroughs (Lambeth, Southwark, Lewisham, and Croydon) and a population of approximately 1.36 million [21, 22]. The Clinical Record Interactive Search (CRIS) system was developed in 2008 to allow researchers to search and retrieve anonymised SLAM EHRs. CRIS accesses a large volume of diverse data, from both structured fields (such as drop-down menus) and de-identified free text (such as progress notes and correspondence), and currently has over 500,000 cases represented in the system. CRIS operates within strict governance framework designed and implemented with service user involvement and is approved as a database for secondary analysis by the Oxford C Research Ethics Committee (18/SC/0372) [21, 22]. This project received input from inception to write-up from service user groups such as the SLAM Service User and Carers Advisory Group [23] and an Expert by Experience Group at the Violence and Abuse Mental Health Network (VAMHN).

### Cohort

We identified all mental health service users aged 16 years or older who were receiving SLAM services between 16.12.2019 and 15.06.2020. Following the declaration of COVID-19 as a public health emergency, UK entered a national lockdown in March 2020, which was characterised by citizens being advised to stay at home. We have used 16.03.2020 as a reference date for when restrictions first began to emerge. We examined all EHRs, using a novel Natural Language Processing (NLP) [24] algorithm, to establish whether service users had a recorded mention of violence victimisation up until 15.06.2020. Victimisation was defined as the recipient of violence (violence is the use of physical force or power, threatened or actual, against another person that results in—or has a high likelihood of resulting in—harm) [25] and included domestic violence, defined as experienced violence (as a victim) between family members, intimate partners, ex intimate partners, and household members. Details on the development of this NLP algorithm have been published [24]. NLP algorithms have more generally been applied extensively in CRIS, allowing the extraction and coding of data from free-text fields (e.g., clinical progress notes), considering the linguistic context in which named entities appear. As opposed to simple keyword search, the violence algorithm seeks to ascertain instances of violence applying to the patient in question, as opposed to violence that has occurred to a family member, for example, or negation statements. The victimization algorithm underwent extensive program development and evaluation, achieving a precision (positive predictive value) of 83% for victimization and 92% for domestic violence specifically and recall (sensitivity) of 93% and 95%, respectively. All active service users were also further distinguished on whether or not they have ever had mentions of victimisation/DV in their records prior to 16.03.2020.

### Outcomes and covariates

We examined three main outcomes and their occurrence in the period following the first COVID-19 lockdown (16.06.20- 1.11.21): acute care; referrals to emergency department liaison services; and all-cause mortality. Acute care was ascertained by establishing whether or not service users had a new inpatient hospital admission and/or Crisis Resolution Team (CRT) episode in SLAM services in the period following the lockdown. In the UK CRTs are used as an alternative to hospital admissions and offer rapid assessment and treatment at home [26]. We used the occurrence of an emergency department mental health services liaison referral as an indicator of a severe level of crisis. Such instances could involve a patient presenting to Emergency Department or a Place of Safety (in the UK this can be in a hospital, police station, emergency department) and this has previously been successfully used as a proxy for self-harm presentations [27]. All-cause mortality was determined using date of death over the observation window available in CRIS via regular monthly updates for SLaM records from the national mortality spine.

Socio-demographic, socioeconomic and service use factors were examined in the period between 16.03.20 and 15.06.20. Socio-demographic factors such as gender, age and ethnicity were ascertained using structured fields in CRIS. Seventeen ethnic group categories were collapsed into “British” (including British, Irish), “Other White” (all other White Backgrounds), “Asian” (Bangladeshi, Chinese, Indian, Pakistani, White and Asian, any other Asian background), “Black African”, “Caribbean”, and “Other” (including: any other mixed background, any other ethnic group or ethnicity not stated), due to small numbers in some individual categories. Socio-economic variables included relationship status and neighbourhood-level social deprivation level based on the Index of Multiple Deprivation, derived by linking addresses to UK Census data providing deprivation data at the lower super output area level (LSOA; a standard administrative unit with approximately 1500 residents). Data on homelessness were also used as an indicator of socio-economic status and categorised as having high level of deprivation. Clinical factors included primary ICD 10 diagnoses recorded in clients’ notes using data from structured and free text. We examined service use over the lockdown period including the number of face-to-face contacts with outpatient services; number of days spent as an in-patient; and number of days active under a Home Treatment Team to help us unpick any potential effect on the associations with the outcomes under investigation.

### Statistical analyses

STATA 13 was used to conduct all statistical analyses. Characteristics were summarised for the total cohort, as well as for all those with/without recorded victimisation during the first lockdown. We also summarised the characteristics of these groups by gender. Having plotted Kaplan–Meier curves with log-rank tests, and following checks of proportional hazard assumptions, Cox regression procedures were used to examine the association between victimisation status and the adverse outcomes. Multivariable models included potential confounders such as age, gender, ethnicity, relationship status, deprivation status, psychiatric diagnoses, and lockdown service use. Statistical models were built in a hierarchical manner to help understand the associations that were observed. In addition, we conducted sensitivity analyses for all outcomes, where we excluded patients from the comparison group with any recorded victimization prior to 16.03.20. Finally, we presented the unadjusted and fully adjusted Cox regression models stratified by gender for all outcomes.

## Results

We identified 21,037 mental health service users aged 16 years or older who received SLAM services between 16.12.2019 and 15.06.2020. In total, 3,610 (17.2%) had victimisation recorded in their clinical case notes between 16.3.2020 and 15.6.2020. Table 1 summarises the socio-demographic, socioeconomic, clinical, and service use characteristics for the entire cohort and by exposure group. Patients with recorded victimisation had a younger mean age, and a higher proportion were women or of Black African ethnicity than the remainder. Patients in this exposure group were less likely to be in a relationship and were more likely to be living in high-level deprivation neighbourhoods. They had a higher frequency of diagnoses related to substance use (ICD-10: F10-19), schizophrenia and other non-mood disorders (ICD-10: F20-29), mood disorders (ICD-10: F30-39) and personality disorders (ICD-10: F60-69), and they had higher service use across all measures.

**Table 1:** Cohort characteristics

Variables	Total cohort	Victimisation during lockdown	No victimisation in the lockdown
Total *n* (%)	21,037	3,610 (17.2)	17,427 (82.8)
Socio-demographic and socioeconomic factors			
Age			
Mean (SD)	42.8 (17.1)	40.0 (16.1)	43.4 (17.2)
Gender (%)			
Female	10,431 (49.6)	1,977 (54.8)	8,454 (48.5)
Male	10,606 (50.4)	1,633 (45.2)	8,973 (51.5)
Ethnic group (%)			
White	8,274 (39.3)	1,367 (37.9)	6,907 (39.6)
Other White	1,746 (8.3)	285 (7.9)	1,461 (8.4)
Asian	614 (2.9)	111 (3.1)	503 (2.9)
Caribbean	1,305 (6.2)	286 (7.9)	1,019 (5.9)
Black African	3,595 (17.1)	838 (23.2)	2,757 (15.8)
Other	5,503 (26.2)	723 (20.0)	4,780 (27.4)
Relationship status (%)			
In a relationship	2,424 (11.5)	344 (9.5)	2,080 (11.9)
Deprivation level in area of residence (tertiles) (%)			
Low level	6,682 (32.8)	934 (26.7)	5,748 (34.1)
Medium level	6,715 (33.0)	1,236 (35.4)	5,479 (32.5)
High level	6,965 (34.2)	1,326 (37.9)	5,639 (33.4)
Clinical factors			
Diagnosis ever recorded (%)			
Organic (ICD-10: F00-09)	1,343 (7.0)	172 (4.9)	1,171 (7.5)
Substance use (ICD-10: F10-19)	3,659 (19.3)	739 (21.1)	2,920 (18.7)
Schizophrenia-like (ICD-10: F20-29)	5,946 (31.1)	1,441 (41.2)	4,505 (28.8)
Mood disorders (ICD-10: F30-39)	5,212 (27.3)	1,166 (33.3)	4,046 (25.9)
Anxiety and other nonpsychotic disorders (ICD-10: F40-49)	4,474 (23.4)	908 (25.9)	3,566 (22.8)
Behavioural syndromes (ICD-10: F50-59)	1,114 (5.8)	176 (5.0)	938 (6.0)
Personality disorders (ICD-10: F60-69)	2,114 (11.1)	709 (20.3)	1,405 (9.0)
Intellectual disability (ICD-10: F70-79)	869 (4.5)	195 (5.6)	674 (4.3)
Developmental disorders (ICD-10: F80-89)	1,398 (7.3)	283 (8.1)	1,115 (7.1)
Face to face contact (Mar’20–Jun’20)			
Mean (SD)	2.1 (5.4)	4.4 (8.7)	1.5 (4.0)
Inpatient bed days (Mar’20–Jun’20)			
Mean (SD)	1.9 (11.5)	8.7 (23.7)	0.4 (5.4)
Home treatment team days (Mar’20–Jun’20)			
Mean (SD)	0.3 (2.6)	1.1 (4.9)	0.1 (1.6)

Table 2 summarises these findings stratified by gender. Briefly, female service users with recorded victimisation were younger than their male counterparts, a higher proportion were in a relationship, and had a diagnosis of a mood or anxiety disorder; females also had a higher proportion of face-to-face contacts but a lower proportion of inpatient bed days. Male service users with recorded victimisation were more likely than females to live in high-deprivation neighbourhoods, and had higher proportions with diagnoses of psychoactive substance use (ICD-10: F10-19), schizophrenia-related disorders (ICD-10: F20-29), intellectual disability (ICD-10: F70-79), and developmental disorders (ICD-10: F80-89).

**Table 2:** Cohort characteristics stratified by gender

Variables	Victimisation during lockdown (*n* = 3,610)		No victimisation in the lockdown (*n* = 17,427)	
	Male (*n* = 1,633)	Female (*n* = 1,977)	Male (*n* = 8,973)	Female (*n* = 8,454)
Socio-demographic and socioeconomic factors				
Age mean (SD)	40.7 (15.5)	39.5 (16.5)	43.5 (16.5)	43.4 (18.0)
Ethnicity group (%)				
White	555 (34.0)	812 (41.1)	3,400 (37.9)	3,507 (41.5)
Other White	121 (7.4)	164 (8.3)	775 (8.6)	686 (8.1)
Asian	42 (2.6)	69 (3.5)	263 (2.9)	240 (2.8)
Caribbean	156 (9.6)	130 (6.6)	570 (6.4)	449 (5.3)
Black African	457 (28.0)	381 (19.3)	1,534 (17.1)	1,223 (14.5)
Other	302 (18.5)	421 (21.3)	2,431 (27.1)	2,349 (27.8)
Relationship status (%)				
In a relationship	113 (6.9)	231 (11.7)	845 (9.4)	1,235 (14.6)
Deprivation level in area of residence (tertiles) (%)				
Low level	404 (25.6)	530 (27.6)	2,845 (32.8)	2,903 (35.5)
Medium level	536 (33.9)	700 (36.5)	2,816 (32.4)	2,663 (32.5)
High level	638 (40.4)	688 (35.9)	3,020 (34.8)	2,619 (32.0)
Clinical factors				
Diagnosis ever recorded (%)				
Organic (ICD-10: F00-09)	80 (5.0)	92 (4.8)	532 (6.5)	639 (8.5)
Substance use (ICD-10: F10-19)	449 (28.3)	290 (15.2)	2,104 (25.9)	816 (10.9)
Schizophrenia-like (ICD-10: F20-29)	871 (54.8)	570 (29.8)	2,833 (34.8)	1,672 (22.3)
Mood disorders (ICD-10: F30-39)	401 (25.2)	765 (40.0)	1,600 (19.7)	2,446 (32.7)
Anxiety and other nonpsychotic disorders (ICD-10: F40-49)	293 (18.4)	615 (32.2)	1,357 (16.7)	2,209 (29.5)
Behavioural syndromes (ICD-10: F50-59)	32 (2.0)	144 (7.5)	206 (2.5)	732 (9.8)
Personality disorders (ICD-10: F60-69)	233 (14.7)	476 (24.9)	573 (7.0)	832 (11.1)
Intellectual disability (ICD-10: F70-79)	118 (7.4)	77 (4.0)	438 (5.4)	236 (3.2)
Developmental disorders (ICD-10: F80-89)	184 (11.6)	99 (5.2)	767 (9.4)	348 (4.6)
Face to face contact (Dec’19–Mar’20)				
Mean (SD)	4.1 (6.7)	4.7 (10.0)	1.6 (3.2)	1.5 (4.7)
Inpatient bed days (Dec’19–Mar’20)				
Mean (SD)	13.0 (28.4)	5.0 (17.9)	0.6 (6.4)	0.3 (3.9)
Home treatment team days (Dec’19–Mar’20)				
Mean (SD)	1.1 (5.0)	1.0 (4.9)	0.1 (1.0)	0.1 (2.1)

Table 3 summarises output from Cox proportional hazards models for the associations between victimisation and all adverse outcomes. In total, 959 (26.6%) service users with victimisation mentioned in their records had evidence of crisis care (i.e., hospitalisation/ CRT episode) between 16.6.2020 and 1.11.2020, compared to 6.6% in the remainder. Overall, 578 (16%) patients with mentions of victimisation had an emergency department mental health referral in the same period, in comparison to 911 (5.2%) of the patients with no mentions of victimisation during the lockdown. Although, adjustments for socio-demographic, socioeconomic, clinical and service use covariates had attenuating effects for both outcomes, the fully adjusted models indicated a significantly elevated risk for acute care (aHR: 2.1, 95% CI 1.9–2.3, *p* < 0.001) and emergency department referrals (aHR: 2.0, 95% CI 1.8–2.2, *p* < 0.001) for service users with mentions of victimisation in their clinical notes over the first lockdown, as compared to service users without such mentions. The strengths of the associations were even more pronounced for both outcomes following sensitivity analyses comparing patients who had mentions of victimisation (over the lockdown) to those who have never had such mention in their entire clinical records.

**Table 3:** Cox regression analyses of the association between victimisation and adverse outcomes

	Victimisation vs. no victimisation in the lockdown	
	HR (95% CI)	*P* value
Acute care models		
Unadjusted model	3.9 (3.5–4.3)	*p* < 0.001
Model^a^	3.9 (3.5–4.3)	*p* < 0.001
Model^b^	3.9 (3.6–4.3)	*p* < 0.001
Model^c^	3.6 (3.2–3.9)	*p* < 0.001
Model^d^	3.5 (3.1–3.9)	*p* < 0.001
Model^e^	2.4 (2.2–2.7)	*p* < 0.001
Model^f^	2.1 (1.9–2.3)	*p* < 0.001
Sensitivity analysis^g^	3.9 (3.0–5.0)	*p* < 0.001
Emergency department referral models		
Unadjusted model	3.5 (3.2–3.9)	*p* < 0.001
Model^a^	3.4 (3.1–3.8)	*p* < 0.001
Model^b^	3.5 (3.2–3.9)	*p* < 0.001
Model^c^	3.1 (2.8–3.5)	*p* < 0.001
Model^d^	3.1 (2.8–3.4)	*p* < 0.001
Model^e^	2.4 (2.1–2.7)	*p* < 0.001
Model^f^	2.0 (1.8–2.2)	*p* < 0.001
Sensitivity analysis^g^	3.9 (2.9–5.1)	*P* < 0.001
All-cause mortality models		
Unadjusted model	1.1 (0.9–1.4)	*P* = 0.281
Model^a^	1.5 (1.2–1.9)	*P* < 0.001
Model^b^	1.1 (0.9–1.4)	*P* = 0.265
Model^c^	1.6 (1.2–1.9)	*P* < 0.001
Model^d^	1.6 (1.2- 2.0)	*P* < 0.001
Model^e^	1.5 (1.1–1.9)	*P* = 0.003
Model^f^	1.5 (1.1–1.9)	*P* = 0.003
Sensitivity analysis^g^	1.8 (1.3–2.6)	*P* = 0.001

^a^Adjusted for age

^b^Adjusted for gender

^c^Adjusted for all socio-demographic factors—age, gender, ethnicity, relationship status

^d^Adjusted for all socio-demographic and socioeconomic factors- deprivation

^e^Adjusted for all demographic, socioeconomic and service use factors- face-to-face contacts; and service use (number of inpatient bed days; number of days active under a Home Treatment Team) in the three months prior to 16/6/20

^f^Adjusted for all demographic, socioeconomic, clinical factors, all service use factors and ICD 10: F20-29; ICD10: F30-39; ICD10: F60-69; ICD10: F70-79

^g^Analysis is excluding patients from the Everyone else group who have ever had mention of victimization prior to 16/3/20

From the patients who had victimisation mentioned in their records during the lockdown, 86 (2.4%) died in the follow-up period in comparison to 396 (2.3%) of patients with no record of victimisation during the lockdown. Adjusting for all factors, especially age, attenuated the overall association. The final adjusted model indicated that patients with victimisation mentioned in their records during the lockdown had a significantly increased risk for death (aHR: 1.5, 95% CI 1.1–1.9, *p* = 0.003). This risk was again even more pronounced when compared to patients who had no recorded victimisation in their entire EHRs. On further examination, we also found that the risk of death was particularly high amongst service users with mentions of victimisation who were aged between 16 and 28 (aHR: 2.8; 95% CI 1.0–7.8; *p* = 0.042, not shown) and aged 55 or over (aHR: 1.6, 95% CI 1.1–2.2, *p* = 0.006, not shown). Table 4 summarises the associations with adverse outcomes stratified by gender. In brief, although we did not observe a statistically significant interaction with gender, after adjusting for possible confounders, men had slightly higher hazard ratios than women for associations with all-cause mortality and emergency department referrals.

**Table 4:** Unadjusted and fully adjusted Cox regression analysis models stratified by gender for all outcomes

	Male HR (95% CI), *p* value	Female HR (95% CI), *p* value
Acute care		
Unadjusted model	4.3 (3.7–4.9), *p* < 0.001	3.6 (3.2–4.2), *p* < 0.001
Adjusted* model	2.0 (1.7–2.4), *p* < 0.001	2.1 (1.8–2.5), *p* < 0.001
Emergency department referral		
Unadjusted model	3.6 (3.0–4.2), *p* < 0.001	3.5 (3.0–3.9), *p* < 0.001
Adjusted* model	2.1 (1.7–2.5), *p* < 0.001	1.9 (1.6–2.2), *p* < 0.001
All-cause mortality		
Unadjusted model	1.3 (0.9–1.8), *p* = 0.121	1.0 (0.7–1.4), *p* = 0.959
Adjusted* model	1.5 (1.0–2.2), *p* = 0.032	1.3 (0.9–1.9), *p* = 0.236

*Adjusted for all demographic, socioeconomic, all service use factors and ICD 10: F20-29; ICD10: F30-39; ICD10: F60-69; ICD10: F70-79

## Discussion

This is one of the first studies to examine the risk of adverse outcomes associated with records of victimisation during the first UK COVID-19 lockdown, amongst mental health service users, using a large mental healthcare dataset from a socially diverse catchment. Our results indicated that service users who had victimisation mentioned in their records during the first COVID-19 lockdown had significantly higher risk for acute care, emergency department referral and death in the year following the lockdown. We found that just over 17% of the cohort had mentions of victimisation in their records during the first lockdown. This is consistent with previous systematic review evidence [28] reporting a pooled prevalence of 18% for physical violence experienced by people with a serious mental illness. Similar to their findings, we also found that the group with mentions of victimisation in their records had a higher proportion of service users from ethnic minority groups, and a larger proportion were living in higher-deprivation neighbourhoods (which included homelessness). Our findings further indicated that patients with recorded victimisation during the lockdown appeared to be more unwell and with a higher level of service use. Given previous evidence [29] of the bi-directional relationship between experiencing violence and mental health, causal pathways may be complex. One possible explanation is that patients who were more unwell were more likely to seek mental health support (consistent with the higher levels of service contact observed) and were therefore asked about experiences of violence as part of a comprehensive assessment [27]. Alternatively, it is possible that those patients with severe illnesses are more likely to have experienced more severe violence and to have disclosed this.

Stratifying the cohort characteristics by gender allowed us to examine differences within the group that had mentions of victimisation. Our findings indicated that female service users who had mentions of victimisation during the first lockdown had a higher proportion of mood and anxiety disorders, behavioural syndromes, and personality disorders. This is in keeping with previous research [30] which has indicated that there is an increased risk for adult lifetime partner violence amongst women with depressive, anxiety disorder, and PTSD. Furthermore, we also detected a higher prevalence amongst women for face-to-face contact with outpatient services as compared to their male counterparts. Two possible hypotheses, not necessarily mutually exclusive, are that there may be inherent differences in the way men and women use mental health services, and/or that victimisation can have an effect on how men and women present to services (e.g., seek support). However, these require further investigation.

In keeping with early research [2, 6] indicating that people with pre-existing mental disorders were reporting increased mental health symptoms following the onset of the pandemic, we found that service users who had victimisation mentioned in their clinical notes during the lockdown period, were at a particularly high risk of hospitalisation or CRT following the lockdown. One possible explanation is that service users were prevented from seeking help earlier as a result of changes in how mental health services were delivered, including cancellation of face-to-face appointments, early discharges, and generally harder to access mental health services [2]. Alternatively, as well as police and third sector evidence that victimisation and domestic abuse increased during the first lockdown, experiences of victimisation may have led to worsening symptoms [2]. Future research needs to focus on exploring possible mechanisms in more detail, including qualitative approaches.

The experience of domestic violence specifically is recognised to be associated with severe outcomes such as suicidal behaviours [28]. Furthermore, domestic violence is prevalent in people presenting to emergency services for self-harm [31]. Previous research [27] using EHRs has successfully used referrals to emergency department as a marker of severe crisis (such as self-harm episode) beyond what is captured by the mental health inpatient admissions and/or CRT referrals. Our results indicated that in the post-lockdown period, service users with recorded victimisation had significantly increased risk of emergency department referral as compared to service users who did not have such mentions in their notes. Existing research has indicated that emergency department mental health support including support for self-harm decreased during the lockdown [27]. One possible explanation is that the need for this population did not decrease, but that patterns of seeking and accessing care changed during and after the COVID-19 lockdown. There is some evidence to support this hypothesis from the UCL COVID-19 Social Study [32], which reported that fewer than 8% of people who reported self-harm or suicidal thoughts, and 10% of people reporting physical abuse, sought mental health professional support during the first few months of the lockdown.

Death is one of the most severe outcomes of domestic violence and victimisation, as a result of suicide, homicide, or physical health problems [27]. From previous research, we know that people with mental health disorders have excess mortality in comparison with the general population [33]. Research [34] investigating death amongst mental health service users following the COVID-19 onset determined that service users had an increased standardised mortality ration before, during and after the COVID-19 outbreak. Our results indicate that a mortality disparity also exists between service users with/without recorded victimisation. Younger (aged 16–28) and older adults (aged 55 +) appeared to be disproportionately affected by this. Unfortunately, due to the small numbers in many of the individual causes of death, we were unable to investigate this in further detail. Future research examining a longer period of time post-COVID lockdown may have the statistical power to investigate specific causes of death which is likely to shed more light on the mechanisms behind these results.

This study had several strengths. SLAM holds one of Europe’s largest EHR registers, accessing diverse and dynamic clinical data recorded during routine secondary mental healthcare. Therefore, the dataset provides a real-life snapshot of what is recorded in patient’s notes when they come into contact with services, providing clinical validity. In addition, the large sample we investigated allowed for sufficient power to investigate outcomes such as all-cause mortality. To detect victimisation and domestic violence we used validated NLP algorithms [24] with good precision and recall, thus capturing most of the instances recorded in the clinical records and reducing the chances of misclassification.

There are several limitations that need to be borne in mind when interpreting the findings from this study. It is possible that the victimisation prevalence we have detected under-represents what is truly occurring in people receiving mental healthcare population- there is consistent evidence that all forms of victimisation and domestic violence are not consistently asked about in routine clinical care [29]. Furthermore, we observed that patients who had victimisation mentioned in their records had much higher service use; therefore, it is possible that victimisation is most often asked about when patients are very unwell. However, most research has found that the severity of mental health problems are more likely to be a consequence rather than a cause of more severe domestic abuse [35]. In addition, residual confounding may have occurred, including confounding by indication as we did not attempt to measure and adjust for the duration of mental disorder or investigate the role of specific symptoms. Lastly, the NLP algorithms we used to detect victimisation did not have sufficient temporal sensitivity; therefore, we were not able to ascertain more concretely the timing of the victimisation event. The temporal limitation was mitigated in several ways. We ensured that all of the participants were in receipt of clinical services in the 3 months before the first lockdown. This allowed us to examine all clinical records for victimisation mentions in the time before they entered the observation period. In addition, we conducted sensitivity analyses, where we removed patients with prior mentions of victimisation from the comparison group.

## Conclusion and implications

In summary, our findings suggests that mental health service users who had mentions of victimisation in their clinical records during the first UK COVID-19 lockdown, were at an increased risk of experiencing adverse outcomes such as acute care, having an emergency department referral and mortality in the year following the lockdown. This study provides further evidence on how mental health service users were faring over COVID-19 period. Future research which examines the mechanisms underlying these associations is needed, to inform how services and care provision should respond if the situation recurs. However, it is clear that patients who are known to be current victims of violence and abuse are at increased risk of relapse and death in the months following such changes in society and mental health services. Some services have not yet returned to their previous ways of working (e.g., remote delivery of interventions is continuing) and this may be risky in assessments of victimisation, particularly if it is domestic, as the perpetrator may be in the home and able to hear the clinical conversation. Bearing in mind this increased risk of subsequent adverse outcomes, it is important that victimisation is identified safely and responded to appropriately [36]. Mental health professionals, therefore, need to ensure that they know how to assess and respond to in this area of patients’ lives, for example, using publicly available guidance (https://www.kcl.ac.uk/mental-health-and-psychological-sciences/research/lara-vp-download-form; https://oxfordhealthbrc.nihr.ac.uk/our-work/oxppl/domestic-violence-and-abuse/).

## Data Availability

The data that support the findings of this study are available on request from the corresponding author (GKS). The data are not publicly available due to the Information Governance framework and REC approval in place concerning CRIS data use.
